# “The Feeling Is What Counts”: Fathers’ Perspectives on Child Risk and Protection within the Ultra-Orthodox Context

**DOI:** 10.3390/ijerph20054385

**Published:** 2023-03-01

**Authors:** Netanel Gemara

**Affiliations:** School of Social Work, Faculty of Social Welfare and Health Sciences, University of Haifa, Haifa 3498838, Israel; ngemara@univ.haifa.ac.il

**Keywords:** context-informed perspective, child risk, child protection, fatherhood, poverty, ultra-orthodox

## Abstract

Context is known to have substantial influence on issues pertaining to child development. Nevertheless, the field of child well-being, risk, and protection is rooted in Western modernized research and experience, often overlooking contextual dissimilarity. The present study aimed to explore risk and protection for children in a distinct context: the Ultra-Orthodox community, which is an insular and religiously close-knit society. Fifteen in-depth interviews with Ultra-Orthodox fathers dealing with issues of child risk and protection were conducted and thematically analyzed. Analysis of the findings revealed two major areas that fathers viewed as posing potential risk for children: poverty and a lack of paternal presence. In both cases, the fathers emphasized that appropriate mediation of these circumstances can diffuse their potential harm. The discussion outlines the different ways fathers proposed mediating potential risk situations, highlighting distinct religion-related methods. It then considers specific, context-informed ramifications and recommendations and notes limitations and directions for future study.

## 1. Introduction

The importance of incorporating contextual elements into the study of child development has been stressed by many child development theories, perhaps most notably the “ecological approach” [1]. Context refers to “a set of circumstances or facts that surround and contribute to the full meaning of an event or situation” [2] (p. 262). Risk factors for children are defined as “experiences, behaviors, characteristics, and contexts that increase the likelihood that a child will experience maltreatment” (p. 2). A protective factor is not merely the absence of a risk factor, but rather a factor that reduces individual risk by moderating the risk in a positive direction [3]. A context-informed approach to child risk and protection “seeks to identify the relevant contexts in the construction of the ‘risk’ and ‘protection’ categories from the perspectives of parents, children, and child protection professionals” [4] (p. 7). This approach highlights contextual realms such as the socio-political, the historical, the economic, the cultural, the religious, and the spiritual, to name a few [5]. The influence of culture and context on risk and protective perceptions combines varied contextual elements, including the child’s attributes, the family, health and sickness, spirituality, and how to react to risk [6]. Nonetheless, the field of child well-being, risk, and protection is rooted in Western modernized research and experience [7]. As a result, the pathways and mechanisms by which culture and context influence the definition, etiology, prevention, and intervention in cases of child maltreatment have remained ambiguous [8].

This article seeks to broaden the context-informed understanding of child risk and protection within the Ultra-Orthodox community. It begins with a review of the issue of non-hegemonic fatherhood and the community under study and then moves to a presentation of the study’s methodology and findings. The article concludes with a discussion and a presentation of the implications for child protection in minority religious societies.

### 1.1. Non-Hegemonic Fatherhood and the Study of Risk for Children

A still-expanding interest that has emerged in the field of fatherhood in the past few decades [9] has given birth to a number of new research and conceptual frameworks [10]. Still, mainstream theories on fatherhood have been based largely on a fatherhood that is middle-class, dominant, and Westernized [11]. Mainstream research tends to overlook the impact of social, racial, and structural inequality on issues of fatherhood [12]. The view of fathers in non-hegemonic societies usually stems from a positivistic and privileged hegemonic perspective that results in the stereotyping of these fathers [13]. A major example of this stigmatic perspective is the literature’s stance on impoverished fathers. A prevalent notion that poverty minimizes a father’s emotional bonding with his children [14] was later challenged by scholars who opposed the culture of poverty discourse and showed the varied patterns of impoverished fathers’ relationships with their children [15]. Adapting intersectional theories [16] was recommended in exploring non-hegemonic fatherhood and advancing a context-informed, culturally competent theorization of the phenomenon [17].

### 1.2. The Ultra-Orthodox Community in Israel

The Ultra-Orthodox community is highly religious and lives according to an all-encompassing system of Jewish “commandments” [18]. In Ultra-Orthodox society–which is collectivistic, maintains strong social ties, and provides mutual assistance [19]–rabbinical figures have a great influence on the lives of individuals and communities as a whole [20]. Ultra-Orthodox families are large and patriarchal and maintain clear gender roles [21]. Their communities tend to segregate themselves from general-secular society by residing in separate cities and attempting to limit their contact with outside parties as much as possible. One result of this dynamic is the underutilization of formal state social services [22]. Central values of the Ultra-Orthodox include believing in God, obeying religious commandments, studying Torah, performing acts of kindness, honoring parents and elders, and practicing modesty [23].

### 1.3. Ultra-Orthodox Socio-Economics

The Ultra-Orthodox community is one of the poorest sectors in Israel, with 52.4% of the population living below the country’s poverty line. Moreover, the gap between the poverty line and the disposable income of a poor Ultra-Orthodox household in Israel is 36% as opposed to 30% among the non-Ultra-Orthodox population, suggesting that the Ultra-Orthodox poor are poorer than their secular counterparts. Poverty in the Ultra-Orthodox community is pervasive and permanent and has worsened over the past decade [24]. A major reason for this situation is the fact that many Ultra-Orthodox men (close to 50%) choose to devote their time to Torah study and religious worship instead of employment [25]. In many households, the woman is the sole breadwinner, and many of the men who do work lack an academic education and therefore receive low wages. In addition, the large number of children in most families contributes to the prevalence of poverty in the community [26].

### 1.4. Childhood in the Ultra-Orthodox Community

Members of the Ultra-Orthodox community regard the bearing of children as an important religious commandment and a primary value. In Israel, Ultra-Orthodox families are, on average, much larger (M = 6.6) than families in the general secular population (M = 2.1) [26]. Children are expected to honor and respect their parents; to demonstrate discipline toward authority figures; to be mature, independent, and responsible; and to delay gratification [27]. The Ultra-Orthodox parenting style tends to be more authoritative than the secular approach [28], and corporal punishment is perceived as normative [29]. Education regarding the observance of religious commandments begins at a very young age. Fathers are generally responsible for the religious aspects of the child’s upbringing, whereas mothers are in charge of the material aspects [30]. Strict gender separation is imposed within the community, meaning that outside the immediate family, boys and girls have no contact with one another [31]. Boys are expected to study Torah and to play a larger and more central role in prayers and community religious gatherings, whereas girls are not obligated to study Torah or to engage in communal prayer. Many girls receive a broader general education than boys (for example, in math, the sciences, and English) and are expected to take part in household chores, including taking care of their younger siblings [27].

### 1.5. Child Risk and Protection in the Ultra-Orthodox Community

Although research on child risk and protection in the Ultra-Orthodox community has thus far been scarce, the literature has addressed a number of unique issues of risk and protection, including religion’s role in risk for children [32,33], interactions between child welfare officers and rabbis [34], sexual abuse [35], stigma [22], and corporal punishment [29]. A unique perception of risk in the Ultra-Orthodox is the concept of “spiritual risk,” which refers to the Ultra-Orthodox parents’ concern regarding a possible decline in what they perceive as their children’s level of spiritual wellbeing [36]. There is a prevalent concern regarding the phenomenon of child maltreatment and abuse within the Ultra-Orthodox community. This is due to several factors, including the under-reporting of instances of child physical [37] and sexual abuse [38], as well as higher rates of corporal punishment [29]. These issues raise significant concerns and warrant further attention and examination.

The present study aims to contribute to the body of knowledge regarding child risk and protection within close-knit religious minority communities by providing an insider’s view of this phenomenon. The study focuses on fathers, whose voices are often silent in the research field of parenting and multicultural parenting [39]. In addition, it sheds light on the understudied Ultra-Orthodox population [4] whose findings may impact our knowledge of other religious minorities. This study was guided by the following question: What are the constructions, perceptions, beliefs, and meanings associated with child risk and protection among Ultra-Orthodox fathers in Israel?

## 2. Materials and Methods

The study employed a context-informed perspective, which examines the role of varying contextual factors, including, among others, the socio-cultural, the historical, the economic, and the religious [4]. The study utilized qualitative methodology aimed at generating a comprehensive and exhaustive understanding of phenomena by exploring experiences, perceptions, beliefs, worldviews, and meanings formed by the interplay of environmental contexts and subjective interpretations [40,41].

### 2.1. The Sample

Fifteen fathers from the Ultra-Orthodox community were recruited. The initial interviewees were selected based on previous acquaintance with the researcher, followed by the use of a snowball sampling method. The two criteria for inclusion were: (a) self-definition as belonging to the Ultra-Orthodox community; and (b) being a parent of at least one child under the age of 18. The ages of the participants ranged from 28 to 45 (M = 35) years. All were married and had between two and seven children (M = 4.87). Forty percent of the fathers in the sample were Avereichim: Ultra-Orthodox men who devote their time fully to religious activities in general and the study of Torah in particular and who do not work. No compensation was provided to the participants. It is essential to underline that the absence of children’s voices in this study is a key issue that must be addressed in future research.

### 2.2. Data Collection

Semi-structured interviews lasting between one and two hours were conducted by the author, an Ultra-Orthodox researcher, and a father of four at locations chosen by the participants. The interview guide that was used corresponded with studies on risk and protection carried out with other minority communities [42] and was adapted for use specifically with the Ultra-Orthodox community. The questions were open-ended, and probing questions were used to elicit additional data. The interview guide dealt with the following areas: (a) perceptions of good care, psycho-social risk, and protection of children; (b) perceptions regarding the definition, etiology, impact, and prevention of child maltreatment, as well as the issue of intervention; and (c) religious aspects of child abuse and neglect.

### 2.3. Data Analysis and Validation

All interviews were audio-recorded and transcribed verbatim in accordance with the method of thematic analysis [40]. Basic familiarity with the data was established, followed by the identification of basic units of meaning and the formation of codes using the process of axial coding. Subsequent links and hierarchies were formulated among and within codes until theoretical saturation was achieved [43].

The study’s trustworthiness was increased through peer debriefing, an audit log, and member checking [44,45]. The researcher analyzed the data independently and then in conjunction with a diverse research group composed of experts in the fields of child maltreatment, multiculturalism, and qualitative methodology, and the varied backgrounds of the different researchers enabled multiple perspectives. In addition, the author’s identity as a member of the studied group provided the study with an insider’s perspective, while other members of the research group provided an outsider’s perspective; each perspective posed both advantages and disadvantages for qualitative research [46]. A detailed documentation of the research process was recorded throughout the different stages of the study [47]. Member checking was conducted during the data collection process, and participants were asked to respond to issues raised in previous interviews. In addition, during the data analysis, interviewees were asked to clarify details that were ambiguous for the researchers [48].

## 3. Results

An analysis of the interviews revealed that the Ultra-Orthodox fathers interviewed perceive the subjective experience of the child as a decisive factor in determining whether a circumstance constitutes risk. From their perspective, the potential risk in certain circumstances can be reduced or avoided by helping the child perceive the situation in a positive light. The two major factors that emerged from the interviews as having the potential to cause risk were poverty and paternal absence. In both cases, the participants emphasized that since the child’s subjective experience is the determining factor for risk assessment, these circumstances should not automatically be perceived as risk factors but should be seen in context.

### 3.1. Between Material Lack and Emotional Abundance

The Ultra-Orthodox community in Israel is one of the country’s poorest sectors as a result of various attributes of many families in the Ultra-Orthodox community, including a large number of children, unemployment, and cultural-religious views. The interviewees referred to various aspects of material lack, including financial difficulties, crowded conditions, a lack of food, and a lack of clothing. All of these conditions were perceived by the interviewees as situations that do not automatically pose a risk for children.

#### 3.1.1. Financial Difficulties

According to Aharon, a father of four, children whose families are contending with financial difficulties should not be assessed according to their socio-economic status but rather according to the child’s subjective feelings.

Let’s say there is a situation of low socio-economic status, the child will not necessarily feel neglected. The parents might tell him, “We do not have much money now, but when we are better able, we will make an effort to get you what you need.” The question is what they communicate to him and whether they can communicate that this is not the whole world. They can minimize it, and in this way, he will not feel as if he has nothing but rather that he has other things. The question is how he feels about that. The feeling is what counts.

According to Aharon, parents play an important role in mediating the family’s difficult financial situation to their children. If they manage to communicate a message that focuses on future change in addition to minimizing the difficulty, the situation does not constitute a risk.

The parents’ ability to manage the family’s impoverished situation was viewed by Jacob, an Avreich and a father of three, as a central component for determining whether a child is at risk due to financial hardship.

Financial difficulties can put a child at risk if the parents do not know how to manage the situation. If at home the child always hears, “We don’t have, we don’t have,” he will go and look for where “there is…” and automatically be at risk. However, if the parents do not emphasize it, or perhaps do not talk about it at all, then the child will not know about it. Once he receives warmth and love, he will not be at risk.

Although Jacob does not deny the potential risk posed by poverty, he maintains that parents either need to not emphasize it or avoid talking about it altogether. In other words, if the parents adopt a stance that frees the child from concern about finances, the child will not be at risk.

A “religious justification” that can be offered to a child to influence his or her subjective experience of poverty was suggested by Israel, an Avreich, and a father of four:

If the parent communicates (to the child) that he is an Avreich and he is miserable—“it is difficult to be a Jew,” as they say—then the child may think that the street is more glistening. However, if while admitting the financial difficulties, the parents communicate that they are happy, and they keep the difficulties between themselves, and they convey in a real way… that they are doing what they are doing happily and out of choice, and that, although it is not always easy, we sacrificed the joys of this world so that daddy can study Torah, not as a tortured saint, but out of joy, the joy of serving God.

The above interviewee’s words suggest that a child can develop resilience by understanding that the lack he is encountering is actually a sacrifice in order to achieve a higher spiritual objective. The transformation of the child’s difficulties into a means of serving God reconstructs the child’s experience, thus eliminating its risk.

#### 3.1.2. Crowding

The large number of children in Ultra-Orthodox families, combined with situations of financial lack, produces crowded living conditions. In the following quote, Judah, a father of four and a teacher in an Ultra-Orthodox school, demonstrates the perspective of the significant role of a child’s subjective experience as opposed to his or her physical surroundings:

I can give you an example of a child; they have 16 children at home. They live in a three-room apartment. Every night they lay down mattresses all over the house, and they collect them in the morning. Nevertheless, I have never encountered such an emotionally well-built child. He receives a lot of support, a lot of warmth, a lot of love, listening, empathy, and compliments. It simply does not affect him, no matter how big the house is or what it looks like. It is a child who is emotionally healthy in a rare way. In contrast, there are children with a lot of money whose parents take them abroad, and they can give them everything, but they live in tension. They do not know how to behave; they live like wounded animals.

Judah is referring to a child who is part of a family with many siblings (16) who live together in a small three-room apartment, which is a condition that, from a professional standpoint, would be considered dire poverty. Nevertheless, in his opinion, the fact that the child’s parents provide him with emotional supplements, such as support, warmth, love, and empathy prevents this difficult situation from impacting the child’s emotional health.

#### 3.1.3. Food

The belief that material lack does not necessarily constitute a risk for a child was shared by Yoav, a principal of a girl’s high school and a father of seven who also serves as a rabbinical lawyer. According to him, material lack can be compensated for emotionally. “There are children without food,” he explains, “and they are not at risk, because the family provides all the emotional nurturing.”

The view articulated by Yoav was articulated in many of the interviews: not only is the emotional realm more significant for the child than the physical realm, but the former can replace the latter, eliminating risk altogether for the child.

An important contextual element that affects a child’s subjective experience of poverty is noted below by Yanki, a father of three:

Look, people say that when a home does not have much food, the children will look outside and search for it. However, every child is accustomed to something different, what he sees as something unique or as a surprise. For a child who knows that his family has no money, buying special chocolate for the holiday is: “Wow!” Every home has its own standards.

Because they compare it to the socio-economic standards of the general secular population, professionals tend to assess the financial reality in the Ultra-Orthodox community as constituting poverty. Nevertheless, Yanki believes that since the known standard in the Ultra-Orthodox community is much lower, the child’s experience is dependent on his or her relative situation. In these circumstances even a small improvement in a child’s familiar standards will give him an elevated feeling.

#### 3.1.4. Clothing

Rafael, a community rabbi, noted the subjective feelings of children and their ability to be influenced by acknowledging their parents’ efforts to meet their needs:

A child does not necessarily need nicer fabric. It depends on the abilities of the parents and the feelings of the child. The price on the tag is irrelevant. A child needs to know that his parents tried the best they could.

According to Rafael, a child needs the actual cloth or the concrete physical object less than he or she needs to feel their parents’ love and dedication, which do not cost money.

A strategy for parental intervention drawn from a traditional source was offered by Moshe, an Avreich. From his perspective, parents can give their child the hope that things will improve in the near future:

If a child is wearing a torn shoe, you can tell him: “Alright, in two or three days we will get to it, we will buy (it for) you…we will not let you walk like this.” You cannot always solve everything right away, but you can give him the feeling that it will be fine. Rabbi Akiva promised his wife Rachel that he would buy her a piece of jewelry. I do not have money now, but I will buy it for you when I am able to. It provides relief. The child knows that he is not officially neglected…– that it is something that can change.

Moshe’s insight on helping a child deal with poverty is drawn from a well-known cultural source: Nedarim 50:2. This section of the Talmud depicts a situation in which Rabbi Akiva and his wife Rachel are in dire financial straits, and Rabbi Akiva comforts his wife by promising her that things will eventually improve. According to Moshe, a similar intervention can be used in a situation in which a child is experiencing poverty.

In sum, this section addressed the issue of poverty, which the medical and psychological literature identifies as an established risk factor for children, and which is a prevalent reality in the Ultra-Orthodox community. The participants addressed different aspects of poverty, including financial struggles, numerous children, a lack of food, and ragged clothing. With regard to all these aspects, the interviewees insisted that the objective reality, which has the potential to cause harm to children, will not affect them negatively as long as their subjective experience is positive.

### 3.2. Between Parental Absence and Independence

The second arena of risk mentioned by the fathers was the lack of parental presence. Children in the Ultra-Orthodox community are afforded greater independence in comparison to similar-aged children in the general secular population, with older children often responsible for taking care of their younger siblings [19]. Some of the participants explained that parents work and study extensively during the week and that their interactions with their children take place primarily on weekends. The interviewees that referred to this potentially disadvantageous situation, and in a similar manner to poverty, again emphasized the importance of the children’s subjective experience when determining the potential risk associated with a lack of parental presence. The potential risk situations to which the fathers referred included child parentification, busy parents, and loneliness.

#### 3.2.1. Child Parentification

The notion that poverty only constitutes a risk if the child’s subjective experience is negative was similarly articulated by the same interviewee, Jacob, with regard to the potential risk posed by the lack of parental presence:

What I have learned is that what counts is whether the child is fine with it. I have a neighbor like this. Every day, the child comes home by himself. He is in the first grade now, but (he did so) even earlier. He asks for the house key, which is with us; he goes home; he is responsible; he gets the other children from (preschool and) kindergarten. The mother can leave the house; she might ask the neighbors to keep an eye on the children. There were times when the baby cried, and my wife went to check, and the child said, “Mother is supposed to come home (soon), it is fine.” They are totally fine with it.

The need of many mothers to go to work, in addition to the large number of children in many families, creates a situation in which older children take care of their younger siblings. This phenomenon, which is widespread around the world and termed sibling caretaking, is also spoken of in the literature as “child parentification.” The practice is often viewed by professionals as a risk factor (Hafford, 2010). Nonetheless, it is a common condition in Ultra-Orthodox homes, and, according to Jacob, it should not be considered a risk factor per se without also considering the child’s subjective experience.

#### 3.2.2. Busy Parents

In situations in which parents are very busy, as suggested by Nissan, a teacher in an Ultra-Orthodox boys’ elementary school, the fact that they are not around much does not automatically pose a risk for their children:

When parents work from morning until night and the children need to prepare meals by themselves and organize the house, this is not neglect if the child sees his father at night and hears two positive words from him, is advised by him, and sees that he is being supervised. True, he is a bit more independent, and he is doing a bit more than other children do, but this is not a problem once the child feels that someone is looking after him and helping him. The child gradually acquires his independence and knows how to do things by himself.

An attentive response to parental absence, according to this father, by providing the child with a secure sense of supervision, can moderate his negative experience. An appropriate approach may even promote the child’s independent development.

An innovative perspective on the role of coping in a child’s upbringing was articulated by Issac, a father of four, who offers the lack of parental presence as an example:

There can be a situation in which the father is engrossed in his work and the mother is engrossed in her stuff, and the parents are, in essence, not involved in the lives of their children. There is food, but that is not enough; they are not involved with their children’s lives, which is extremely important in my opinion. Nevertheless, I myself was given an education stressing the need to struggle. A child needs to understand that even if it is difficult, the world will not come to an end. It gives him tools for life. Struggling is part of life. If a child does not learn how to cope, he will not know how to deal with difficulty when he gets older.

In the above quote, Isaac articulates a fundamental outlook on life: as he views it, life is an experience of challenge and struggle. As it is important to provide children with tools to deal with such hardships, challenging experiences are an essential part of a child’s upbringing.

#### 3.2.3. Loneliness

The value of coping with life in a child’s upbringing was also mentioned in the context of loneliness. According to Avi, an Avereich, a lack of parental presence should not necessarily be perceived as a risk for children and in fact can be recognized as a constructive condition:

In my experience, when a young child stays at home by himself on a regular basis in the evening and does not mind it, the family might claim that it is better for the child. It matures him. It gives him responsibility.

This interviewee asserts that hardships may be perceived as constructive for a child’s development, as long as certain circumstances exist, for example, when the child is not disturbed by them.

Similarly, Daniel, a father of seven, explains his views regarding a child who does not need to struggle:

A child at risk is a child who does not know how to cope with issues in life. Every person goes through difficulties. It can be being alone, medical issues, or financial issues. There is no one who does not experience these kinds of issues. That’s how life is. If you approach life with the understanding that you have a role to play and that you need to play your role regardless of the difficulties, then you can overcome any difficulty. You need confidence in yourself and in God; you need to know that you have been assigned a role in the world, that your role is awaiting you and you alone, and that there is nobody else to take your role–the role forever remains yours alone.

Daniel feels that when the child is presented with a religious justification for his burden, it will not constitute a risk for him. In his eyes, connecting the child to his spiritual role may transform the difficulty into a building block in the child’s mental and religious resilience.

This subsection addressed a potential risk factor that is found in the Ultra-Orthodox community: a lack of parental presence. The interviewees addressed different aspects of this phenomenon, including child parentification, busy parents, and loneliness. Although these aspects have the potential to create risk for children, the participants insisted that by intervening and mediating these circumstances, thereby influencing the subjective experience of the child, he or she will stay protected from risk.

## 4. Discussion

The findings highlight two conditions that the fathers noted as posing potential danger to children and that child protection professionals commonly identify as constituting risk: (1) poverty [49,50], and (2) a lack of parental presence [51,52]. The interviewees, however, insisted that these two conditions do not necessarily always pose a risk for children. As they see it, the determinant of risk should be the subjective experience of the child in question. Poverty and lack of parental presence, the participants maintained, do not pose a risk per se when these circumstances are moderated, thus influencing the child’s subjective experience.

### 4.1. The Child’s Subjective Experience

Further analysis of the interviews revealed two main approaches explained by the fathers with regard to the centrality of the subjective experience of the child in cases of potential adversity. One approach deals with the ability of the parents to influence and construct the child’s subjective experience. The second reflects a cultural-ideological perception of the role played by coping in a child’s development, which transforms the perception of risk for children in potentially risky circumstances.

#### 4.1.1. Parental Intervention

The participants argued that parents can help their children develop a resilient subjective experience under adverse conditions in both an indirect and direct manner. Indirect reinforcement includes approaches that do not openly address the difficulties being experienced by the child and the family by concealing them and not talking about them. Participants also stated that giving a child plenty of positive affection balances out the difficulties and eliminates risk. Finally, the fathers held that when children become aware of their parents’ immense efforts to meet their needs, a lack of attention does not affect them negatively. Direct interventions include minimizing the difficulty facing the child and helping him or her focus on the positive aspects of their life. In addition, the fathers suggested supporting the child by telling him or her that things will become better in the future. Lastly, interviewees advised explaining to children that the difficulties they are experiencing are essentially a sacrifice for a higher spiritual and religious cause, thus making the pain more bearable and less harmful.

#### 4.1.2. A Coping Perspective

The second approach articulated by participants offers an innovative perspective on the child’s hardships; it holds that facing difficulties is a vital part of a child’s upbringing. One of the interviewees stated that a child who does not experience obstacles while growing up is essentially “a child at risk.” This unique perspective caused some of the interviewees to maintain that situations such as poverty, lack of parental presence, and others that professionals regard as posing risk to children are not only *not* dangerous for children but rather can be viewed as valuable contributors to a child’s healthy development. This perception is based both on the kind of upbringing the interviewees experienced and on religious sources.

#### 4.1.3. Context-Informed Perspective

The unique perception of child risk and protection presented in this article shifts the focus from the child’s objective reality to the child’s subjective experience. This perception can be understood in light of the context-informed perspective, which highlights the significant role of environmental context in studying individual development [1,4] and warns of the danger of assessing a child’s wellbeing in accordance with universalistic and individualistic standards. The idiosyncratic context of the Ultra-Orthodox community creates circumstances of potential risk. Varied societal, cultural, and religious elements make the Ultra-Orthodox community one of the poorest communities in Israel [26]. Poverty, large families, religious devotion, and other factors may contribute to the lack of parental presence [53,54], as reported by the participants. The distinct perception of child risk and protection presented in this article suits this unique contextual environment, which is a finding that also emerged from a study on working poor Ultra-Orthodox women [55]. Cognitive reframing, a psychological mechanism that involves ascribing a constructive interpretation to a given situation, has been identified as an effective buffer against stressors [56]. In an environment that contains inherent objective “risk factors,” it is well understood how a perception of protection that minimizes the objective reality and intensifies the importance of the subjective realm of the child’s experience is constructed. This context may also encourage the construction of a perspective that views the child’s struggle as an essential developmental component. Additional research on other contexts with inherent physical “risk” potential should be conducted in order to determine the nature of the interplay between context and risk and protective construction and how additional cultural elements (for example, religious attitudes regarding spirituality as taking precedence over physical needs) may play a role in this construction process. The proposed approach is consistent with the arguments of other researchers who caution against using an individualistic approach to the study of poverty resilience instead of a structural context-based perspective [57].

#### 4.1.4. Religious-Cultural Mediating Strategies

Some of the interviewees mentioned cultural-religious mediating strategies for affecting the child’s subjective experience. Such interventions are noteworthy because of their substantial proximity to the family’s world of reference [58]. The use of religious beliefs as a means of coping has been reported as helpful in coping with a variety of stressors, including physical illnesses [59], stress, depression [60], and trauma [61]. In the present study, one traditional source mentioned by the participants is a well-known Talmudic legend recounting classical scholar Rabbi Akiva’s way of coping with his family’s poverty. Another religion-based intervention is the interviewee’s suggestion to frame poverty and the family’s struggle as a sacrifice for their religious way of life. Finally, one father mentioned reminding children of their assigned spiritual roles as a way of helping them cope with their struggles. Further research is required in order to assess the effectiveness of such religious interventions.

#### 4.1.5. Impoverished Fatherhood

The findings of this article challenge the common view in the literature that perceives poverty as a risk for fatherhood [13]. The interviewees demonstrated that impoverished fathers are able to develop innovative ways of maintaining a relationship with their children and of protecting them from the potential negative effects of poverty. The specific context and culture of the Ultra-Orthodox community impact not only the notion of poverty [32,62] but also the construct of fatherhood. These findings may promote further research on the issue of non-hegemonic fatherhood.

### 4.2. Implications

The findings of this study lend further credence to the importance of professionals taking into account the context in which a child lives when assessing child risk. The participants in this study were convinced that conditions that professionals view as risk contributors are not necessarily dangerous for children once contextual elements, such as the role of the parents and the cultural-religious attitude toward coping, are taken into account. On the contrary, some of the interviewees felt that such circumstances might even contribute to a child’s wellbeing. This communal stance challenges risk assessment practices within the Ultra-Orthodox community and other minority groups. Future research is recommended to determine similar mediation strategies in other minority communities.

In addition, the study findings may provide professionals with additional culturally informed ways of promoting resilience in children from the Ultra-Orthodox community who face poverty, a lack of parental presence, and other adversities. Special attention should be paid to culture-based interventions in accordance with traditional sources, as such interventions are closely linked to cultural values and, as such, may be more appropriate.

The perspective of Ultra-Orthodox children on these mediating strategies is an essential component that is not addressed by this study. Further research is required to determine whether the perception articulated by the fathers resembles the experience of Ultra-Orthodox children. Additionally, recommended is an exploration of the effectiveness of this approach in enhancing resilience. Moreover, implementation of the ideas presented in this article must proceed with caution in order to prevent justification of child neglect under the guise of multicultural validation. Professionals are cautioned not to automatically consider a behavior abusive when it is performed in a different culture. However, erroneously accepting a problematic action because it is performed in a cultural context may contribute to child abuse [63].

Future research endeavors should aim to address several significant areas that remained partially explored in the current study. This includes a thorough examination of the role played by the community in the protection of children as well as the extent of utilization of formal welfare services provided by the state by the Ultra-Orthodox community. Furthermore, this study did not delve into crucial facets of the child’s experience, such as the ramifications of any discrepancies that may exist between the child’s expectations and the realities of the family. Additionally, the study did not examine the distinction between religious and other reasons for the father’s absence or its impact on the child’s experience. Moreover, the socio-economic status of the child’s peers may also have a bearing on the child’s experience. Finally, it is important to note that family dynamics, such as the family’s birth order, may also play a role in shaping the child’s experience.

### 4.3. Limitations of the Study

The sample in this study may have been influenced by a sample bias stemming from the purposive sampling method, as is the case in many qualitative studies. An essential limitation of the study is the lack of the child’s perspective. The interviewed fathers’ conceptualization of their children’s experience needs to be assessed in future studies that bring the voice of children though their experience of potentially harmful circumstances. In addition, the interview guide did not exclude participants from addressing both male and female children; yet the fathers mostly discussed risk and protection in reference to their sons, whereas their daughters were rarely mentioned. A future study must incorporate the experiences of both daughters and mothers. Furthermore, the tendency of minority group members to provide a positive image of their community (i.e., social desirability bias) hinders their capacity for self-reflection regarding the influence of their behavior on their children. Therefore, their reports should be viewed with caution [64]. The interviewer’s identity as part of the Ultra-Orthodox community poses both advantages and potential limitations for the study [45].

## 5. Conclusions

This study utilized qualitative methodology to explore Ultra-Orthodox fathers’ perspectives on children’s risk and protection. The fathers emphasized the parents’ vital role in mitigating the child’s experience under challenging circumstances, such as poverty and a lack of parental presence. This study contributes to the context-informed child protection field by enriching the outlook of policymakers, professionals, and parents regarding ways of providing resilience for children facing complex circumstances. In addition, the fathers’ accounts provide an essential viewpoint in the field of fatherhood by highlighting the involvement and commitment impoverished fathers have in their attempt to provide a ‘good enough’ fatherhood.

## Data Availability

Not applicable.
